# CD3e-immunotoxin spares CD62L^lo^ Tregs and reshapes organ-specific T-cell composition by preferentially depleting CD3e^hi^ T cells

**DOI:** 10.3389/fimmu.2022.1011190

**Published:** 2022-10-26

**Authors:** Shihyoung Kim, Rajni Kant Shukla, Hannah Yu, Alice Baek, Sophie G. Cressman, Sarah Golconda, Ga-Eun Lee, Hyewon Choi, John C. Reneau, Zhirui Wang, Christene A. Huang, Namal P. M. Liyanage, Sanggu Kim

**Affiliations:** ^1^ Department of Veterinary Biosciences, The Ohio State University, Columbus, OH, United States; ^2^ Department of Microbial Immunity and Infection, The Ohio State University, Columbus, OH, United States; ^3^ Division of Hematology, The Ohio State University, Columbus, OH, United States; ^4^ Department of Surgery, University of Colorado Denver Anschutz Medical Campus, Aurora, CO, United States; ^5^ Infectious Disease Institute, The Ohio State University, Columbus, OH, United States

**Keywords:** CD3e immunotoxin, tissue-resident T cells, regulatory T cells, tolerance induction, T-cell lymphoma therapy

## Abstract

CD3-epsilon(CD3e) immunotoxins (IT), a promising precision reagent for various clinical conditions requiring effective depletion of T cells, often shows limited treatment efficacy for largely unknown reasons. Tissue-resident T cells that persist in peripheral tissues have been shown to play pivotal roles in local and systemic immunity, as well as transplant rejection, autoimmunity and cancers. The impact of CD3e-IT treatment on these local cells, however, remains poorly understood. Here, using a new murine testing model, we demonstrate a substantial enrichment of tissue-resident Foxp3+ Tregs following CD3e-IT treatment. Differential surface expression of CD3e among T-cell subsets appears to be a main driver of Treg enrichment in CD3e-IT treatment. The surviving Tregs in CD3e-IT-treated mice were mostly the CD3e^dim^CD62L^lo^ effector phenotype, but the levels of this phenotype markedly varied among different lymphoid and nonlymphoid organs. We also found notable variations in surface CD3e levels among tissue-resident T cells of different organs, and these variations drive CD3e-IT to uniquely reshape T-cell compositions in local organs. The functions of organs and anatomic locations (lymph nodes) also affected the efficacy of CD3e-IT. The multi-organ pharmacodynamics of CD3e-IT and potential treatment resistance mechanisms identified in this study may generate new opportunities to further improve this promising treatment.

## Introduction

CD3e-IT, a fusion of the catalytic domain of diphtheria toxins with the CD3e-binding portion of a CD3e antibody is a promising precision medicine for an effective T-cell depletion. CD3e-IT has shown preclinical and clinical efficacy for the treatment of T-cell lymphomas (1), autoimmune diseases (2) and transplant rejection (3–7), and is currently under evaluation as a preconditioning regimen for cell therapy. A recent *Resimmune* ^®^ [A-dmDT390-bisFv(UCHT1)] (8) clinical trial showed notable response rates of 47–74% in patients with intermediate-stage (stage-IB/IIB or mSWAT score < 50) cutaneous T-cell lymphomas (CTCL) (9). Its effects on stage III/IV CTCL and peripheral blood T-cell lymphomas, however, seemed limited for reasons that remain unclear. The limited efficacy of immunotoxins has been attributed to several potential factors, including pre-existing antibodies or immunogenicity against CD3e-ITs, the lower levels of CD3e or insensitivity of lymphoma cells in advanced CTCL, the lack of penetration of the immunotoxins into target cell sites, and protective tumor microenvironment (9–13). The impact of pre-existing antibodies or immunogenicity against the “second generation” recombinant CD3e-IT seemed to be mild and insignificant (14–16). Other factors that may influence CD3e-IT efficacy include the differential expression of the TCR/CD3 antigen receptor complexes (17, 18), the CD3e isoforms in the TCR/CD3 complexes (19, 20), the polymorphism of CD3e (21), and other cell-intrinsic factors of basically every step of toxin-mediated cell-killing processes (22). The impact of these cell-intrinsic and local factors on *in vivo* CD3e-IT efficacy remains unclear.

CD3e-IT has also proven effective in inducing long-term allograft acceptance in nonhuman primates and swine models (3–7, 23–29). Mounting evidence points to the critical roles of regulatory T cells (Tregs) in inducing tolerance in various treatment settings (30–33), but their roles in CD3e-IT-mediated tolerance remain unclear. CD3e-IT has shown to be more effective in depleting T cells in the lymph nodes (LN) and skin than antibodies (34, 35) and reliably prolonged allograft survival (36, 37). CD3e-IT alone or in combination with a short-term immunosuppressive chemotherapy, however, often showed limitations in sustaining graft function or survival (36, 38–40). Recent CD3e-IT-mediated donor hematopoietic stem cell transplant studies have demonstrated promising results where long-term acceptance of allografts seemed to be finally achievable through mixed chimerism in monkey and swine models (4, 5, 29). Unlike the similar studies utilizing anti-T-cell antibodies (41–43), there was however no clear evidence that T-cell anergy or regulation by Tregs serve as the dominant mechanism of allograft tolerance. The CD3e-IT-mediated transient chimerism, instead, facilitated the robust and stable humoral immune modulation of donor-specific antibody responses (44). Treg infiltration into an allograft has been reported in a miniature swine model, suggesting a potential local regulatory component (4). Nevertheless, the survival and roles of Tregs in CD3e-IT-mediated tolerance induction remain unclear.

This study presents a detailed pharmacodynamic evaluation of CD3e-IT treatment with a particular focus on the survival of Tregs in different organs. Tissue-resident T cells rarely circulate and instead remain stably parked in the tissue parenchyma of local organs (45–48). These local T cells collectively account for the largest T-cell subset in the body. They have been shown to participate in protection from infection and cancer and are associated with allergy, autoimmunity, inflammatory diseases and transplant rejection (45). Our understanding of these local cells in CD3e-IT treatment settings remains surprisingly poor. Tregs must home to LNs to establish antigen-specific T-cell tolerance (49–51). The activated effector Tregs in LNs may then exert their suppressive functions both locally and systemically. Local Tregs showed a marked skewing of T-cell receptor (TCR) usage by anatomical location which contribute to shaping the unique peripheral Treg population in different secondary lymphoid organs (52). Follicular T regulatory cells (Tfr) that primarily reside in B-cell follicles are critical in shaping humoral immune responses by controlling follicular T helper cells (Tfh) and B cells in the germinal center (53, 54). Like Tfr, other tissue-resident Tregs in nonlymphoid organs or in allografts also play pivotal roles in organ-specific functions and homeostasis (55). Treg infiltration into the graft also coincided with the metastable tolerance achieved in animals and humans (38, 56, 57). Nevertheless, the impact of CD3e-IT on local tissue-resident Tregs or T cells in general remain largely uncharacterized. Only a few recent studies, including our own, have shown differential depletion efficiencies for tissue-resident T cells by anti-T-cell mAb and CD3e-IT treatments in a few selected organs (35, 46). Most past studies have focused almost exclusively on total peripheral blood, lymph node and bone marrow cells in animal models (34, 37, 58–60) and total peripheral blood in humans (1), without drawing any clear distinction between circulating and tissue-resident populations.

Despite the numerous advantages over other animal models, mouse models have been used only rarely for CD3e-IT studies, primarily due to the limited efficacy and toxicity of first-generation murine CD3e-ITs (61). We have recently developed a new murine-version CD3e-IT (S-CD3e-IT) whose safety and treatment efficacy profile is comparable to those of the “second-generation” recombinant CD3e-ITs (46). Here, we demonstrate the significant enrichment of Forkhead box transcription factor (Foxp3) Tregs in the murine model following CD3e-IT. We used the new murine testing model to systemically characterize Foxp3+ Treg enrichment in multiple organs – including the peripheral blood, spleen, lung, Peyer’s Patches, 5 types of lymph nodes (mesenteric, inguinal, mandibular, mediastinal, and lumbar), thymus, and bone marrow – using intravascular staining techniques to analyze circulating and tissue-resident cells separately (46–48). We found S-CD3e-IT effectively depletes both circulating and tissue-resident T cells, but organ-to-organ variations were evident. S-CD3e-IT preferentially depleted CD4+ T-cell subsets that express high levels of CD3e (CD3e^hi^), sparing T-cells with low levels of CD3e (CD3e^dim^). Differential surface expression of CD3e molecules among T-cell subsets, as well as organ-to-organ variations in CD3e expression, drive Treg enrichment and reshape organ-specific T-cell composition. As a result, effector Tregs (CD62L^lo^) with the CD3e^dim^ phenotype were substantially enriched in the tissue-resident pools of secondary and nonlymphoid organs.

## Results

### New murine CD3e-IT effectively depletes tissue T cells with notable organ-to-organ variations

**Figure 1:**
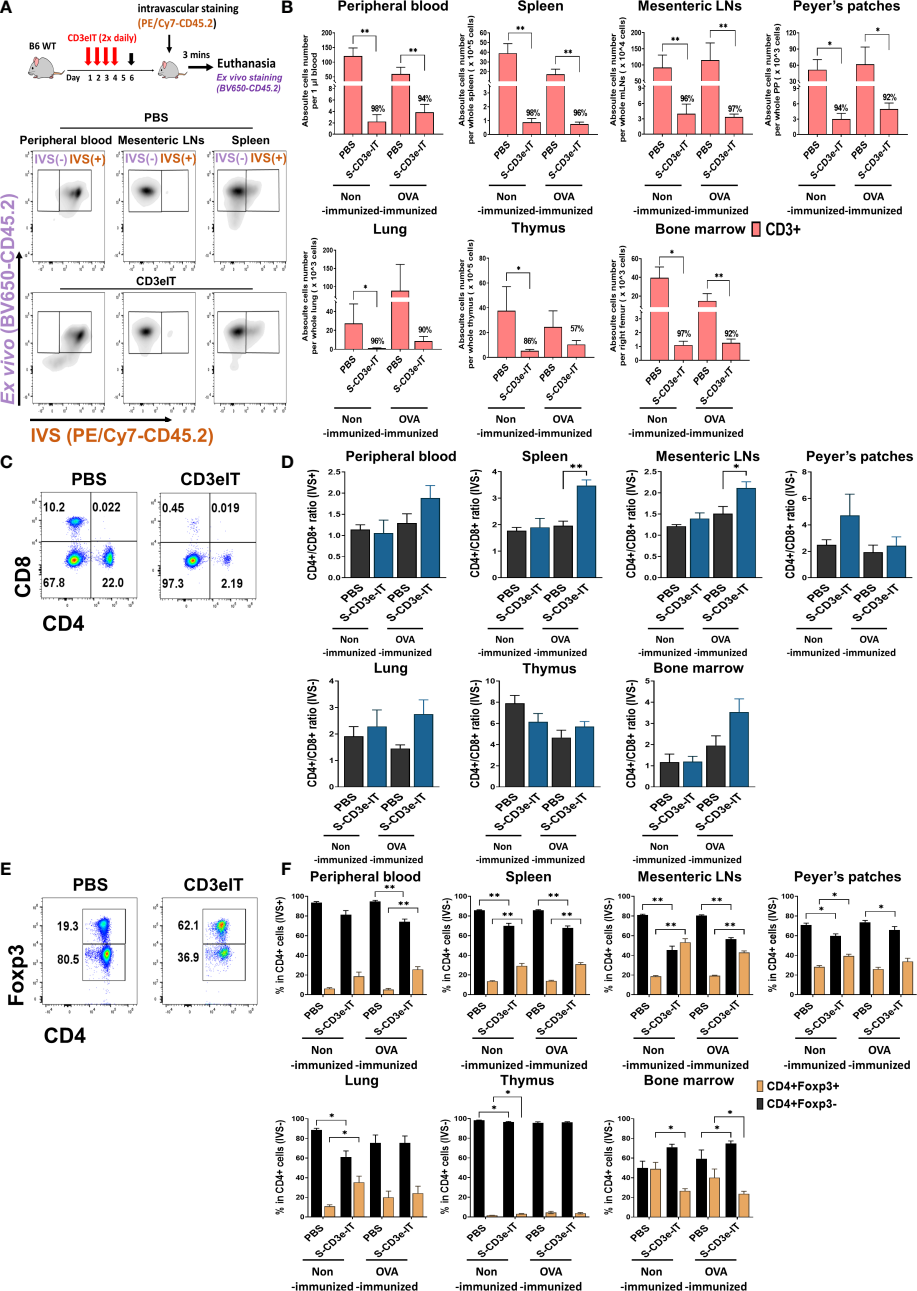
CD4+Foxp3+ Tregs cells enrichment following S-CD3e-IT treatment. **(A)** The diagram shows an S-CD3e-IT treatment (Upper panel) and intravascular staining strategy (Lower panel) to analyze the effect of S-CD3e-IT efficacy on circulating and different Tissue-resident T cells. The flow cytometry gating strategy shows circulating (IVS+) and tissue-resident cells (IVS-) separation in peripheral blood, mesenteric LNs, and spleen. **(B)** The absolute number of CD3+ cells (y-axis) is shown for the peripheral blood (IVS+) and tissue-resident (IVS-) portions of the spleen, mesenteric LNs, Peyer’s patches, lung, thymus, and bone marrow. Absolute cell numbers were calculated based on CountBright Absolute counting beads and total cell counts per organ. **(C, D)** Flow cytometry for CD4+ and CD8+ T cells **(C)** and the ratios of CD4+ to CD8+ for all tested organs **(D)** are shown. **(E, F)** Flow cytometry for CD4+Foxp3- and CD4+Foxp3+ T cells **(E)** and % of these cells in CD4+ cells **(F)** are shown for all tested organs. Nonimmunized mice treated with PBS (*n* = 4~5, depending on organs), or S-CD3e-IT (*n* = 4~6), OVA-immunized mice treated with PBS (*n* = 3~4), or S-CD3e-IT (*n* = 7~8), were compared. (*p < 0.05 and **p < 0.01).

For a detailed pharmacodynamics analysis of CD3e-IT, we employed a new murine testing system that we developed recently (**Figure 1A**) (46). Anti-murine-CD3e-ITs (S-CD3e-ITs) were generated by conjugating streptavidin-saporin (Advanced Targeting System) with non-mitogenic CD3e-mAb (145-2C11 with Fc-silent™ murine IgG1, Absolute Antibody) as described previously (46). S-CD3e-IT was highly precise and efficacious in depleting both circulating and tissue-resident T cells in mice (**Figure 1B**). We tested S-CD3e-IT in both nonimmunized and OVA-immunized C57BL/6J mice. The S-CD3e-IT dosage was chosen based on our previous *in vitro* and *in vivo* dosage study (46). Non-immunized laboratory mice that live in specific pathogen-free (SPF) facilities are known to have limited GC formation (62–64) as well as follicular T cells (65, 66) and CD69+ T cells (67). A systemic immunization induces T-cell activation and infiltration into uninflamed tissues, including both secondary and nonlymphoid organs (45, 65, 67–69). The formation of the germinal center in the spleen and mesenteric LN following the intraperitoneal injection of OVA in complete Freund’s adjuvant was confirmed by immunohistochemistry (**Supplementary Figure 1**). Follicular T cells were detectable in all tested organs (see **Figures 4C**, **5E** below). Akin to previous reports (70), the LN cells of OVA-immunized mice showed a notable increase in surface expression of CD69 (a marker of tissue residency) on CD4+ T cells compared to those in nonimmunized mice (**Supplementary Figure 2A**).

Both nonimmunized and OVA-immunized C57BL/6J mice were then treated with S-CD3e-IT (15mg twice daily by retro-orbital injection) for 4 consecutive days, as described previously (46). Given the high T-cell-specificity of S-CD3-IT (46), the overall pharmacodynamics of S-CD3-IT were evaluated using phosphate-buffered saline (PBS)-treatment as a non-treatment control in this study. On Day 6, these mice were subjected to intravascular staining [IVS; by retro-orbital injection of anti-CD45.2 mAb (PE/Cy7-CD45.2)] followed by euthanasia 3 minutes later. Total organ cells were then *ex vivo* stained with an antibody pool containing another anti-CD45.2 mAb (BV650-CD45.2) (**Figure 1A**). The circulating cells in the vasculature were thus identified as IVS+ and the tissue-resident cells in the tissue parenchyma at the time of injection as IVS−. As expected, in all PBS-treated groups, >99% of peripheral blood CD45+ cells were IVS+, whereas about 98% of LN- and thymus-isolated cells were tissue-resident (IVS−;**Supplementary Figure 3**) (47). Consistent with previous reports (46, 47), >20% of lung-isolated cells and 70-80% of spleen- and BM-isolated cells were IVS− (**Supplementary Figure 3**).

T-cell depletion rates were >90% for both IVS+ and IVS- pools of all tested organs (except for the thymus and a few local LNs), with notable organ-to-organ variations (**Figure 1B** and **Supplementary Figures 3B**, **4A**). The low depletion rates in the thymus may reflect the low CD3e expression of thymocytes as well as the functional properties of the thymus, which was continuing to produce new CD3+ T cells in these mice (see **Supplementary Figure 5**) (71, 72). Different LN types showed highly variable depletion rates depending on the anatomic sites of LNs (discussed below in more detail; see **Figure 5** and **Supplementary Figure 4**). Both CD4+ and CD8+ cells were effectively depleted (**Supplementary Figures 3C**, **4D**). Unlike the reduced CD4/CD8 ratios in CD3-mAb treatment (18), however, the CD4/CD8 ratios in CD3e-IT-treated mice increased in most organs (**Figures 1C, D** and **Supplementary Figure 3B**), indicating preferential depletion of CD8+ T cells over CD4+ T cells by CD3e-IT (**Supplementary Figures 3C**, **4D**) (40, 46). Although T-cell depletion rates were slightly lower in OVA-immunized mice, the organ-to-organ variations were generally consistent between the nonimmunized and OVA-immunized groups (**Figure 1B**).

### Tissue-resident CD4+ FoxP3+ Tregs were enriched in both secondary lymphoid and non-lymphoid organs

The fraction of Foxp3+ Tregs in PBS control mice moderately varied among secondary lymphoid and non-lymphoid organs, appearing in average 5.2% to 28.3% of CD4+ T cells (**Figures 1E, F**), consistent with previous reports in humans and mice (73–77). S-CD3e-IT treatment significantly enriched Foxp3+ Tregs in the tissue-resident pool (IVS−) of most organs, but not in the bone marrow (**Figure 1F** and **Supplementary Figure 4C**). Bone marrow is a known reservoir for Tregs, maintaining strikingly high levels of local Tregs under homeostatic conditions by actively, rather than passively, retaining these cells *via* CXCL12/CXCR4 signals (75). The reduced bone marrow Treg fraction – from 40.1-49.0% (in PBS mice) to 23.8-26.7% (in S-CD3e-IT-treated mice) – may indicate some perturbation of the bone marrow’s ability to actively retain Tregs in its parenchyma (**Figure 1F**). All other organs, however, showed varying levels of the enrichment of CD4+ FoxP3+ Tregs in local tissue sites after S-CD3e-IT treatment. Notably, the Foxp3+ Tregs accounted for nearly half of the total CD4+ T cells survived in LNs (**Supplementary Figure 4C**), an anatomic site orchestrating Treg activities in local organs (52, 78).

### Preferential depletion of CD3^hi^ T cells drives enrichment of Tregs with the CD3e^dim^ phenotype

**Figure 2:**
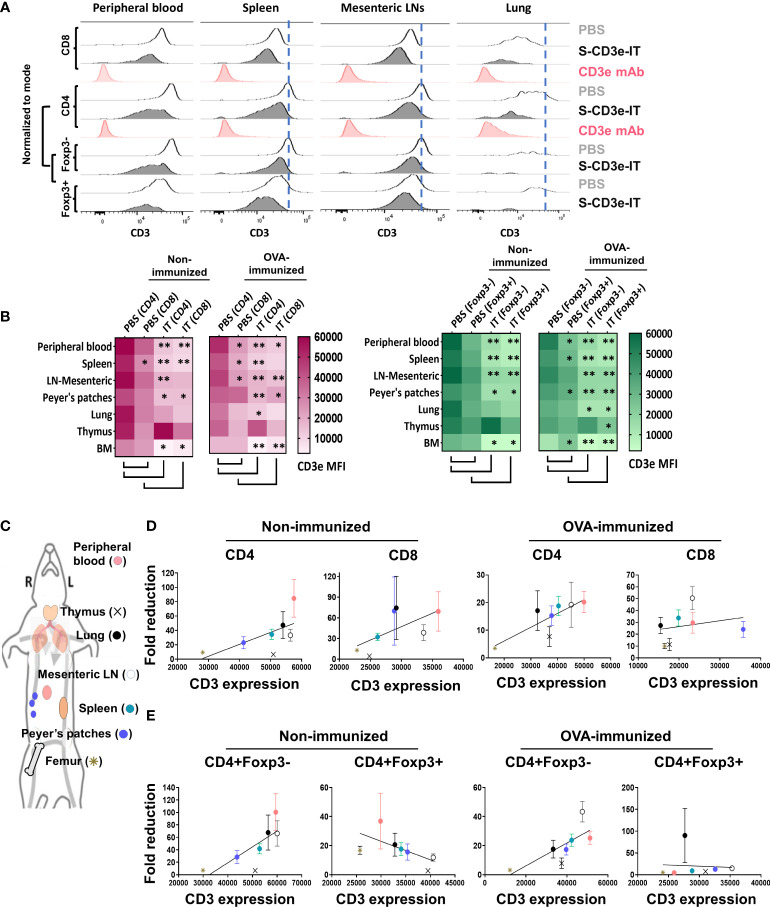
Differential CD3e on T cells drive an enrichment of CD3^dim^ Tregs with organ-to-organ variations. **(A)** CD3e expression (x-axis) on the CD8+, CD4+, CD4+Foxp3-, and CD4+Foxp3+ cells (y-axis, normalized to mode) are shown for the peripheral blood and tissue-resident portions (IVS-) of spleen, mesenteric LNs, and lungs. **(B)** Heatmaps show CD3e mean fluorescent intensities (MFI) for CD4+, CD8+, CD4+Foxp3- and CD4+Foxp3+ cells of all tested organs. Statistical comparison of CD3e MFI on two groups, CD4 (PBS) *vs*. CD8 (PBS) and PBS-treated *vs*. S-CD3e-IT-treated mice, is denoted with an asterisk (*) on the heat map. (*p < 0.05 and **p < 0.01). **(C)** The diagram shows the symbol of all tested organs in **(D, E)**. **(D)** A positive correlation between the fold reductions of CD4+ or CD8+ cell numbers in different organs after CD3e-IT treatment (y-axis) and CD3e MFI of these cells in PBS-treated animals (x-axis). CD4+ and CD8+ lymphocytes for the peripheral blood (red), and tissue-resident (IVS-) portions of the spleen (green), mesenteric LNs (white), Peyer’s patches (purple), lung (black), thymus (cross), and bone marrow (asterisk) are compared. **(E)** A positive correlation between the fold reductions of CD4+Foxp3- cell numbers in different organs after CD3e-IT treatment (y-axis) and CD3e MFI of these cells in PBS-treated animals (x-axis). Note that this correlation was not evident for CD3+Foxp3+ T regs. Nonimmunized mice (PBS *n* = 4~5, and S-CD3e-IT *n* = 4~6, depending on organs); OVA-immunized mice (PBS *n* = 3~4, and S-CD3e-IT *n* = 7~8, depending on organs).

In CD3e-mAb treatment settings, Tregs have been shown to have a survival advantage over other T-cell subsets, likely due to the relatively low surface expression of CD3e molecules on CD4+ FoxP3+ Tregs compared to those of other T-cell subsets (17, 18). The TCR/CD3 complexes on the surface of Tregs are also shown to be enriched in CD3e isoforms with an undegraded N terminal, which probably further contributes to the resistance of these cells to CD3 antibody-mediated cell death (19, 20). Consistent with these reports, CD4+Foxp3- T cells in PBS control mice showed the highest levels of CD3 expression on the cell surface, whereas CD4+Foxp3+ cells showed the lowest levels among tested T-cell subsets (**Figures 2A, B**). Unlike CD3e-mAbs that work through TCR/CD3 modulation (46, 79), however, the binding and internalization of CD3e-IT with CD3/TCR would likely kill the host cells instead of activating TCR/CD3 signals (46). Even a single molecule of ribosomal inactivating toxins, once internalized into a cell, can kill the cell by catalytically inhibiting protein synthesis (80). We found that S-CD3e-IT treatment preferentially depleted T cells that display high levels of CD3e molecules (CD3e^hi^) on the cell surface – reflecting the controlled CD3e-IT dosage in our experiments – and spared T cells with a low-CD3e-expression phenotype (denoted as CD3^dim^) (**Figures 2A, B** and **Supplementary Figure 6**). The CD3^dim^ phenotype was clearly distinct from the CD3e modulation phenotype observed in the CD3e-mAb treatment; CD3e-mAbs almost completely removed or internalized CD3e molecules on the surface of T cells without killing the cells (**Figure 2A**, and **Supplementary Figure 6**) (46, 79). CD8+ T cells display relatively lower CD3e molecules on the surface compared to CD4+ T cells; nevertheless, unlike CD3e-mAb treatment (18), CD3e-IT preferentially depleted CD8+ T cells over CD4+ T cells (**Supplementary Figures 3C**, **4D**) (40, 46) due possibly to the presence CD4+ T-cell subsets resistant to CD3e-ITs.

### Organ-to-organ variations in CD3e expression correlates to differential T-cell depletion rates indifferent organs

In PBS control mice, we also found notable organ-to-organ variations in the CD3e expression levels for each T-cell subset. Peripheral blood T cells and mesenteric LN-resident (IVS-) T cells consistently showed highest levels of CD3e mean fluorescent intensity (MFI) for nearly all T-cell subsets (except CD8+ T cells and Foxp3+ Tregs), whereas bone marrow-resident T cells consistently showed lowest (**Figures 2C–E**). Notably, these differential CD3e MFI levels among tested organs were positively correlated with the depletion rates for CD4+ cells, CD8+ cells, and CD4+FoxP3- cells in these organs in S-CD3e-IT treated mice (**Figures 2D, E**). However, such a pattern reflecting the preferential depletion of T cells that display higher CD3e was not evident with CD4+FoxP3+ Tregs (**Figure 2E**). This discrepancy suggests that, while CD3e-IT still preferentially depletes CD3e^hi^ Tregs over CD3e^lo^ Tregs, the organ-to-organ variations in the depletion of Tregs may be affected by other factors besides CD3e expression levels, including the intrinsic differences in the content of TCR/CD3 complexes (e.g. CD3e isoforms) and the relative insensitivity to external stimuli among Tregs in different organs (17–20). Both the quantitative and qualitative variations in surface CD3e on T cell subsets, therefore, at least partially drive the observed variations in T-cell depletion and Treg enrichment in different organs after S-CD3e-IT treatment.

### CD62L^lo^ Tregs are enriched in secondary lymphoid and non-lymphoid organs

**Figure 3:**
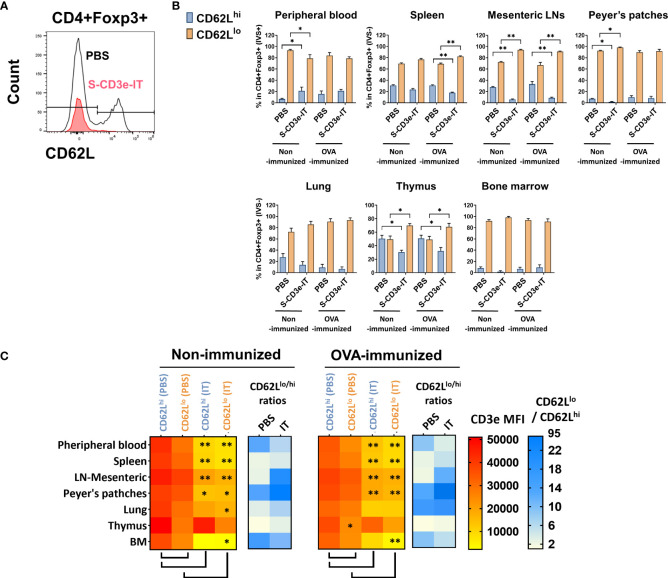
CD3^dim^CD62L^lo^ Treg enrichment correlates with CD3e expression. **(A)** The flow cytometry gating strategy for CD4+Foxp3+CD62L^lo^ and CD4+Foxp3+CD62L^hi^. **(B)** CD62L^lo^ and CD62L^hi^ (% in CD4+Foxp3+ cells) are shown for the peripheral blood (IVS+) and tissue-resident (IVS-) portions of spleen, mesenteric LNs, peyer’s patches, lungs, thymus, and bone marrow. **(C)** The heatmap of CD3e MFI (yellow to red) on CD4+Foxp3+CD62L^lo^ and CD4+Foxp3+CD62L^hi^ cells in tested organs. CD3e MFI on CD62L^lo^ was higher than CD62L^hi^ in all tested organs. A statistical comparison of CD3e MFI of two groups, CD62L^lo^ (PBS) *vs.* CD62L^hi^ (PBS) and PBS-treated *vs.* S-CD3e-IT-treated mice, is denoted with an asterisk (*) on the heat map. The heatmap of the CD62L^lo^-to-CD62L^hi^ ratios (white to blue). The ratio increased after S-CD3e-IT treatment except in peripheral blood and bone marrow. Nonimmunized mice were treated with PBS (*n* = 4~5, depending on organs) or S-CD3e-IT (*n* = 4~6). OVA-immunized mice were treated with PBS (*n* = 3~4) or S-CD3e-IT (*n* = 7~8). (*p < 0.05 and **p < 0.01).

The L-selectin (CD62L) is a crucial lymphoid homing molecule. Treg populations can be largely classified as CD62L^hi^ and CD62L^lo^ Tregs. CD62L^hi^ Tregs are mostly naive and quiescent, whereas CD62L^lo^ Tregs are comprised of recently activated and short-lived cells (81, 82). Both CD62L+ and CD62L- Tregs have shown a similar suppressive capacity for T-cell activation *in vitro* (83, 84), but the CD62L+ Tregs – not the CD62L- counterparts – are likely the major population that provides long-lasting immune tolerance, protecting against lethal acute graft-versus-host disease (85, 86) and delaying diabetes in prediabetic nonobese diabetic mice (84). Studies showed marked increase in CD62L+ Tregs in CD3e-mAb-treated mice (87, 88). By contrast, in S-CD3e-IT-treated mice, we found that CD62L^hi^ Tregs were preferentially depleted by S-CD3e-IT in all test organs, except peripheral blood and bone marrow (**Figure 3**). As expected, the surviving Tregs were mostly the CD3^dim^ phenotype, except those in the thymus (**Figure 3C**). CD62L^hi^ Tregs (mostly CD44^lo^) also showed notably higher CD3e expression on the cell surface than CD62L^lo^ Tregs (mostly CD44^hi^) in PBS control mice (**Figure 3C**), consistent with a previous report that demonstrated a relatively higher CD3e expression on CD44- T cells than those of their CD44+ counterparts (18).

### Proliferation potentials of CD3e^dim^ CD4+ Foxp3+ T cells *vs.* their Foxp3- counterparts

**Figure 4:**
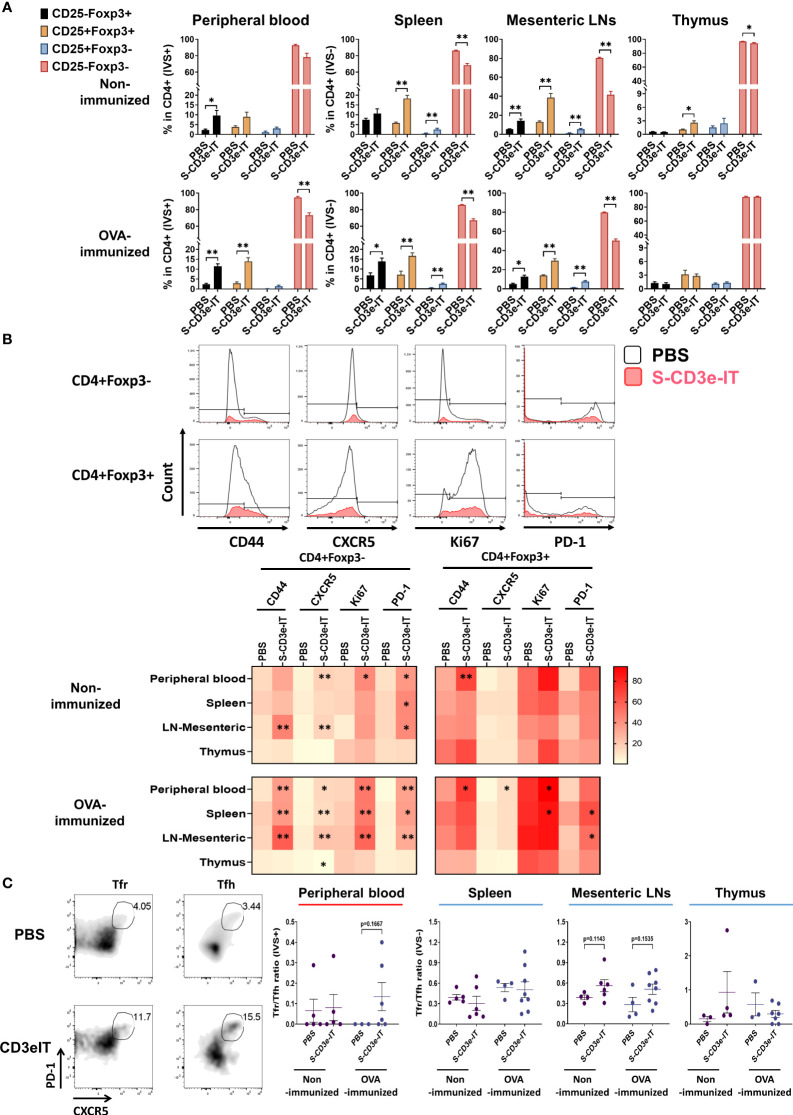
Changes in the functional phenotype of T cells after CD3e-IT treatment. **(A)** S-CD3e-IT treatment enriched CD25+ Tregs cells. CD25+ population (% in CD4+ cells) is shown for the peripheral blood and tissue-resident (IVS-) portions of spleen, mesenteric LNs, and thymus in PBS and S-CD3e-IT treated mice. **(B)** The flow gating strategy is shown to define CD44+, CXCR5+, Ki67+, and PD-1+ cells in CD4+Foxp3- and CD4+Foxp3+ cells (Upper panel). The heat map shows four functional phenotypic changes (light red to dark red) of CD4+Foxp3- and CD4+Foxp3+ T cells following S-CD3e-IT treatment (Lower panel). Statistical comparison between PBS-treated *vs.* S-CD3e-IT-treated mice is denoted with an asterisk (*) on the Heat map. **(C)** The flow cytometry gating strategy is shown to define CXCR5+PD-1+ Tfr or Tfh. The CXCR5+PD-1+ Tfr-to-CXCR5+PD-1+ Tfh ratios are shown for peripheral blood and tissue-resident (IVS-) portions of the spleen, mesenteric LN, and thymus. Nonimmunized mice were treated with PBS (*n* = 4~5, depending on organs) or S-CD3e-IT (*n* = 4~6). OVA-immunized mice were treated with PBS (*n* = 3~4) or S-CD3e-IT (*n* = 7~8). (*p < 0.05 and **p < 0.01).

CD3e-mAb treatment has yielded a transient increase in the relative fraction of Tregs – though not necessarily an increase in Tregs’ cell count – during the early stage of tolerance induction (18, 79, 88–95). In CD3e-IT treated mice, the fraction of CD25+ Tregs were also significantly enriched within the tissue-resident (IVS−) pools of the secondary lymphoid and non-lymphoid organs (**Figure 4A**). Expression of CD25 (an α chain of the high-affinity IL-2 receptor) indicates potential sensitivity to IL-2 and lymphopenia-mediated induction, the primary modulators for the development and maintenance of Tregs (96–98). Given that Tregs, particularly CD62L^lo^ effector Tregs, require TCR/CD3 activation to proliferate and maintain their functional properties (99–101), we next tested the activation and proliferation status of the surviving Tregs – mostly CD3e^dim^ CD62L^lo^ phenotype –by evaluating Ki67, CD44, CXCR5, and PD-1 expression. Our S-CD3e-IT treated mice demonstrated a marginal increase in Ki67 expression (a cell proliferation marker) for CD4+Foxp3+ Tregs in most organs (**Figure 4B** and **Supplementary Figure 7**). CD44, CXCR5, and PD-1 expression also showed only marginal increase in CD3e-IT-treated mice. By contrast, the CD4+Foxp3- counterparts showed a significant increase in CD44+ phenotypes and a 2- to 6-fold increase in Ki67 expression in peripheral blood and secondary lymphoid organs (**Figure 4B** and **Supplementay Figure 7**). Peripheral blood CD4+ Foxp3- T cells in OVA-immunized mice showed a >20-fold increase in PD-1 expression, a marker of exhausted T cells (**Figure 4B** and **Supplementary Figure 7**). Extensive homeostatic expansion of memory T cells has been a common event following CD3e-IT treatment (102).

### Tfr-to-Tfh ratios in mesenteric lymph nodes

**Figure 5:**
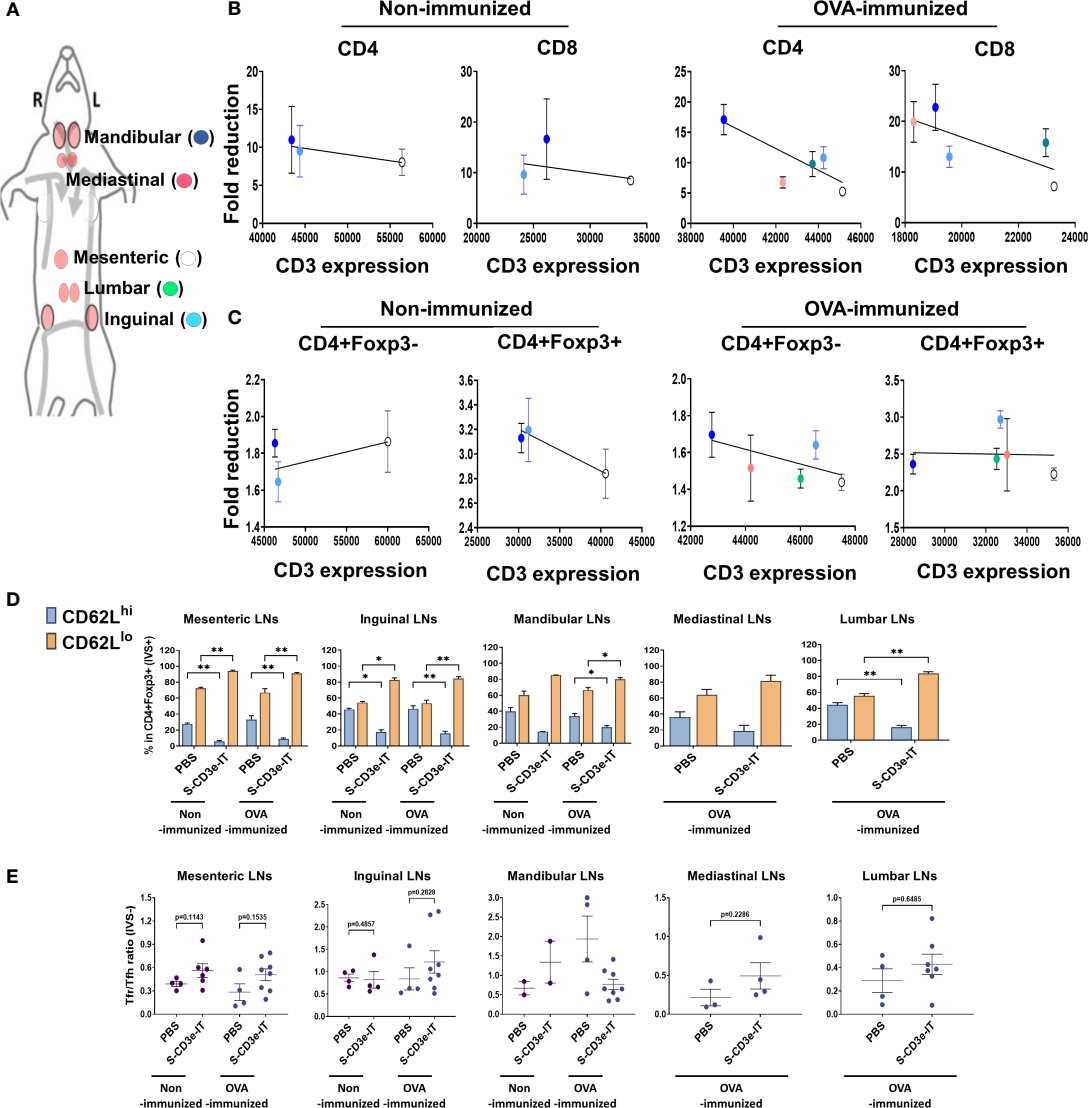
Consistent LN-to-LN variations. **(A)** Symbols of mesenteric (white), inguinal (light blue), mandibular (blue), mediastinal (red), and lumber (green) LNs are shown. **(B)** Fold reductions of CD4+ or CD8+ T cells (y-axis; S-CD3e-IT/PBS) and CD3e MFI of these cells in each LNs (x-axis) are shown. There was no positive correlation between CD3e MFI and CD4+ and CD8+ cell depletion rates. **(C)** Fold reductions of CD4+Foxp3- or CD4+Foxp3+ T cells (y-axis; S-CD3e-IT/PBS) and CD3e MFI of these cells in each LNs (x-axis) are shown. There was no positive correlation between CD3e MFI and CD4+Foxp3- cells. **(D)** CD62L^lo^ and CD62L^hi^ (% in CD4+Foxp3+ cells) are shown for these LNs. The surviving cells were enriched in CD4+Foxp3+ cell pools with the CD62L^lo^ phenotype. **(E)** The Tfr-to-Tfh ratios (% in CD4+) are shown for these LNs. S-CD3e-IT-treated mice show higher Tfr-to-Tfh ratios in most of these LNs. Nonimmunized mice were treated with PBS (*n* = 2~4, depending on LN types) or S-CD3e-IT (*n* = 2~6). OVA-immunized mice were treated with PBS (*n* = 3~4) or S-CD3e-IT) (*n* = 4~8) *p < 0.05 and **p < 0.01.

We examined the Tfr-to-Tfh ratios in circulation as well as in tissue-resident cell pools. Tfr play a critical role in controlling the Tfh and B-cells involved in donor-specific humoral immunity (53, 54). Recent studies have suggested that reduced Tfr-to-Tfh ratios in circulation are a risk factor for allograft dysfunction or failure (103, 104). In our short-term follow up, we found no notable change in Tfr-to-Tfh ratios in peripheral blood (**Figure 4C**). For the tissue-resident (IVS−) compartments, the spleen and thymus showed no notable change in Tfr-to-Tfh ratios. Despite these organ-to-organ variations, it is noteworthy that most LN types (except mandibular) showed a marginal increase in the Tfr-to-Tfh ratio in OVA-immunized mice after S-CD3e-IT treatment (see **Figure 5E** below). Consistent to these results, previous CD3e-IT-mediated chimerism studies showed an effective and durable modulation of donor-specific humoral responses, while the modulation of donor-specific T-cell immunity was not evident (34, 38).

### LN-to-LN variations in T-cell depletion and Treg enrichment

Last, we evaluated LN-to-LN variations in drug efficacy. LNs are major lymphoid organs located in many parts of the body, orchestrating for local Treg-mediated immune regulation in a tissue-specific manner (52, 78). Studies have shown that drug efficacy in different LNs is highly variable and dependent upon the anatomic sites of the target LNs and drug administration routes, resulting in inconsistent responses to drug regimens (105–107). Consistent with these reports, we observed considerable LN-to-LN variations in T-cell depletion depending on the type of LNs (**Supplementary Figure 4A**). We isolated mandibular, inguinal, mesenteric, lumbar, and mediastinal LNs and compared T-cell depletion in these organs (**Supplementary Figure 4A**). Unlike the other organs tested in this study, there was no correlation between CD3e expression levels and CD4+ or CD8+ T-cell depletion rates among these LN types (**Figures 5A, B**). The T-cell depletion rates in these LNs appear to depend on the anatomic positions of these LNs, rather than CD3e expression levels. Mesenteric LN T cells, for example, consistently showed highest CD3e MFI levels for all T-cell subtypes, while mandibular LN T cells consistently showed lowest CD3e MFI among all tested LN types (**Figures 5B, C**). Regardless of the differential CD3e expression levels among LNs, however, mandibular LNs consistently showed highest depletion rates for most of T-cell types tested and mesenteric LNs showed lowest depletion rates for the most of T-cell subtypes (**Figures 5A–C**). These results are consistent with previous reports addressing the variability of drug efficacy depending on the injection and circulation routes for different LNs (105–107). Interestingly, however, while the Treg composition (CD62L^hi^ and CD62L^lo^) was notably varied in each type of LN, we found all LN types showed a substantial enrichment of CD3^dim^CD62L^lo^ Tregs, accounting for >80% of the surviving Tregs, after S-CD3e-IT treatment (**Figure 5D**). Most LN types, except mandibular LNs, showed an insignificant, marginal increase in the Tfr-to-Tfh ratio in OVA-immunized mice after S-CD3e-IT treatment (**Figure 5E**). These results, therefore, suggest additional layers of treatment resistance and the potential need for treatment optimization to control CD3e-IT efficacy in different types of LNs.

## Discussion

Successful application of precision anti-T-cell biologics requires a detailed understanding of both disease etiology and the *in vivo* efficacy of the drugs in use. This study demonstrates an extensive pharmacodynamics analysis of CD3e-IT treatment using a new murine testing system. Our mouse model enabled a detailed evaluation of CD3e-IT-mediated T-cell depletion in multiple organs for both circulating and tissue-resident cells, evaluated separately. Differential CD3e expression among differing T-cell subsets, as well as organ-to-organ variations in CD3e expression, were identified as a main driver for Treg enrichment and the reshaping of organ-specific T-cell composition after CD3e-IT treatment. Differences in CD3e expression on the surface of CD4+ and CD8+ T cells, as well as Tregs are consistent with those of previous murine and human studies (18, 108). We also found that Tregs, particularly the CD62L^lo^ effector subset, display relatively low levels of CD3e on the cell surface; this difference perhaps provides a survival advantage for CD62L^lo^ effector Tregs. Tregs are also known to have higher amounts of CD3e isoforms with an undegraded N terminal and to be relatively insensitive to antibody stimuli (19, 20); these factors likely bolster their resistance to CD3e-IT-mediated cell death as well.

Notably, we also found substantial organ-to-organ variations in T-cell depletion. Our systemic comparison of 11 different organs – including peripheral blood, spleen, Peyer’s patches, lung, thymus, bone marrow and 5 different LN types (mesenteric, inguinal, mandibular, mediastinal, and lumbar) – identified (i) variations in CD3e expression among tissue-resident T cells of different organs as a major factor contributing to the CD3e-IT-mediated reshaping of local T-cell composition. The organ-to-organ variations in CD3e expression may reflect the unique immunologic status of tissue-resident local T cells mostly controlled by local stimuli, maintaining a distinct TCR/CD3 repertoire (45, 55). Additional factors we identified that contribute to the organ-to-organ variations in T-cell depletion include (ii) the unique functional properties of each organ (e.g. the thymus producing new T cells and the bone marrow actively retaining Tregs), and (iii) the dependency of anatomic locations of LNs in CD3e-IT efficacy which is due likely to the effects of drug injection and circulation routes (105–107). All these factors (i – iii) may collectively affects CD3e-IT treatment efficacy in local organs and the reshaping of local T-cell immunity following CD3e-IT-treatment. The 11 different organs we analyzed here were the maximum number of organs we could reasonably analyze in our current experimental settings. To conduct a systemic comparison of these organs from the same animals, we strategically excluded the skin, lungs, and guts, as each of these organs would have required hours of additional cell-isolation procedures. The depletion of tissue-resident T cells in the lungs and skin have been demonstrated previously (35, 46). Although these studies and our own preliminary data for gut T-cell depletion (data not shown) suggest similar levels of T-cell depletion in these organs, a systemic comparison of all of these organs would allow for a complete understanding of the impact of CD3-IT treatment and immune system recovery following the treatment.

Mounting evidence points to the critical roles of Tregs in promoting tolerance in various treatment settings (30–33). Yet Tregs can act like a double-edged sword insofar as they also suppress anti-cancer T-cell immunity, making the local environment more favorable for cancer growth (55). Despite the crucial need, Treg enrichment and their functional properties in CD3e-IT treatment remained unclear. Similar to the transient enrichment of Tregs in the early tolerance-induction period of CD3e-mAb (145-2C11) treatment in mice (18, 79, 88–95), we also found a substantial enrichment of Tregs after S-CD3e-IT treatment. The phenotypic properties of the surviving Tregs in S-CD3e-IT-treated mice were, however, distinct from those in CD3e-mAb-treated mice. Unlike CD3e-mAb – which induces, not kills, Tregs to lead to active tolerance mechanisms involving CD62L^hi^ Tregs (18, 84–88) – CD3e-IT preferentially kills CD3e^hi^ T cells and enriches CD62L^lo^ Tregs with the CD3e^dim^ phenotype. The CD62L^lo^ effector Tregs with CD3e^dim^ phenotype may have a lower tolerogenic potential than CD62L^hi^ Tregs because, first, CD62L^hi^ Tregs, not the CD62L^lo^ counterparts, are reported to be the major population that provides long-lasting immune tolerance (84–86), and second, CD62L^lo^ effector Tregs require constant CD3 activation to proliferate and maintain their functional properties (99–101). The surviving Tregs with the CD3e^dim^ CD62L^lo^ phenotype were only marginally activated in CD3e-IT treated mice. Given the critical importance of Treg functions during the early tolerance-induction period, this difference in the Treg phenotype may have an important implication for CD3e-IT-mediated transient chimerism studies (29, 44). We also observed an insignificant, marginal increase in Tfr-to-Tfh ratios in most LNs following CD3e-IT treatment; this increase may partly explain the robust and durable humoral immune modulation of donor-specific antibody responses in these transient chimerism studies (29, 44). Treg-mediated donor T-cell tolerance was not evident in swine or monkey models (29, 44). Longitudinal tracking of the Tregs and investigation of B-cell responses in long-term induction settings using our new mouse testing model may prove useful in further investigating these importance of Treg-mediated donor-specific T-cell and humoral tolerance induction.

Compared to antibody-mediated T-cell depletion, CD3-ITs have shown a superior ability to deplete tissue-resident T cells in skin (35), as well as spleen, lung, and mesenteric. LNs (46) This is likely due to different cell-killing mechanisms in these two types of depletion agents; immunotoxins kill a cell through the inhibition of protein synthesis (76), whereas antibodies require an environmental support, e.g. neutrophils, to deplete T cells (35, 102).Previous Alemtuzumab therapy for CTCL patients showed inefficient depletion of skin-resident T cells, including Foxp3+ regulatory T cells (35, 109), whereas CD3-IT [A-dmDT (390)-bisFv(UCHT1)] showed effective depletion of skin-resident T cells in human foreskin-grafted humanized mice (35). The survival rate of skin-resident Foxp3+ regulatory T cells following CD3-IT, however, remains unknown. Given the limited treatment efficacy of Resimmune in treating advanced CTCL (9), understanding and improving the depletion of skin-resident T cells, particularly Tregs, is of significant importance.

CD3e-immunotoxins are a promising short-term, precision treatment option for various therapies that require effective depletion of T cells. Vascular leak syndrome (VLS) has been the major dose-limiting toxicity, but the development of the “second generation” recombinant CD3e-ITs has significantly improved safety profiles in clinical and preclinical studies (1, 14, 16, 110, 111). The recent identification of VLS-inducing (x)D(y) motif of toxin molecules offers new opportunities to further reduce VLS (111–117). Despite the significant improvement, the treatment efficacy of CD3e-IT has been often limited by unknown causes. The new insights gained in this study into potential treatment resistance mechanisms of CD3e-ITs and mechanisms of Treg enrichment in different organs can serve as a new foundation on which to further improve this promising precision medicine in clinical and preclinical studies.

## Methods

### Mice

Male C57BL/6J mice were obtained from the Jackson Laboratory (Bar Harbor, ME). All mice in this study were maintained by the guidelines of the Ohio State University (OSU). 4~6 months old male mice were used in this study.

### S-CD3e-IT preparation

To generate murine version of S-CD3e-IT, Fc-silent CD3e monoclonal antibody (S-CD3e-mAb; Absolute Antibody, Upper Heyford, UK) firstly was biotinylated as previously described (46). Anti-murine version of S-CD3e-IT was prepared by conjugating biotinylated-S-CD3e-mAb with streptavidin-ZAP (Advanced Targeting System, San Diego, Calif) in a 1:1 molar ratio as previously described (46).

### Immunization

For immunizing mice, male C57BL/6J mice were received intraperitoneally with ovalbumin (OVA) emulsified in Freund’s complete adjuvant (CFA) (- 2 weeks of the initiation of S-CD3e-IT) followed by OVA emulsified in Freund’s incomplete adjuvant (IFA) one week after the first OVA/CFA injection.

### *In vivo* experiments

Male C57BL/6J mice were injected into retro-orbital sinus with 15 μg S-CD3e-IT in sterile 200 μl PBS twice a day for four consecutive days and were euthanized on day 6 as previously described (46). On the day of euthanasia, mice were injected into the retro-orbital sinus with a total of 3 mg of PE/Cy7-CD45.2 (104, BioLegend) in 200 μl sterile DPBS. After 3 minutes of injection, the mice were euthanized following the previously established protocols (47, 118). The peripheral blood was collected from the heart. The spleen, five LNs (mesenteric, inguinal, mandibular, mediastinal, and lumber LNs), lung, thymus, and bone marrow (right femur) were harvested, and tissues were processed to isolate leukocytes following previous studies with modifications (119, 120).

### Flow cytometry

The collected leukocytes from peripheral blood, spleen, five LNs, lung, thymus, and bone marrow were incubated with Fc-blocking TruStain FcX™ (anti-mouse CD16/32, BioLegend) antibody, followed by Zombie aqua (Live/dead indicator, BioLegend). The cells were stained with fluorescence-labeled antibodies. The following antibodies were purchased from BioLegend: BV650-CD45.2 (clone 104), PE/Cy7-CD45.2 (clone 104), PE/Dazzle594-CD69 (H1.2F3), Alexa Flour 700-CD3e (500A2), Pacific Blue-CD4 (RM4-5), BV570-CD8 (53-6.7), BV711-CD19 (6D5), PerCP/Cy5.5-CD44 (IM7), APC/Cy7-CD62L (MEL-14), Alexa Flour 647-CXCR5 (L138D7), BV421-PD-1 (RMP1-3D), BV785-CD25 (PC61), and BV605-Ki67 (16A8). PE/Cy5-Foxp3 (FJK-16s) was purchased from Invitrogen. All samples were acquired on a Cytek Aurora, and the data were analyzed with FlowJo software (BD Bioscience). The absolute number of leukocytes was calculated using CountBright™ as previously described (46).

### Foxp3 staining

Staining the transcription factor, Foxp3, was carried out using the Foxp3/Transcription Factor Staining buffer set (Invitrogen, Waltham, MA, USA) and according to the recommended manufacturer manual.

### Statistics

Data represent Mean ± SEM. Mann-Whitney test and 2-way ANOVA using Prism 9 software (GraphPad Software, La Jolla, CA) was used to analyze if there were significant differences between PBS-treated and S-CD3e-IT-treated mice. P value less than 0.05 was considered significant.

## Data availability statement

The original contributions presented in the study are included in the article/**Supplementary Material**. Further inquiries can be directed to the corresponding authors.

## Ethics statement

The animal study was reviewed and approved by OSU Animal Care and Use Committee.

## Author contributions

SaK and ShK designed all experiments. ShK, RS, AB, HY, SC, HC, SG, and G-EL carried out the animal experiment. ShK, AB, and SC performed flow cytometry. SaK and ShK analyzed the data. SaK provided the reagents. SaK and ShK wrote the manuscript. ZW, CH, JR, and NL provided critical appraisal of the manuscript. All authors contributed to the article and approved the submitted version.

## Funding

This research was funded by the National Institutes of Health (NHLBI R00HL116234, NHGRI R01HG010318, and NHGRI R21HG010108; American Society of Hematology Scholar Award to SaK; and C. Glenn Barber Fund to ShK.

## Conflict of interest

The authors declare that the research was conducted in the absence of any commercial or financial relationships that could be construed as a potential conflict of interest.

## Publisher’s note

All claims expressed in this article are solely those of the authors and do not necessarily represent those of their affiliated organizations, or those of the publisher, the editors and the reviewers. Any product that may be evaluated in this article, or claim that may be made by its manufacturer, is not guaranteed or endorsed by the publisher.
