# Supplementary material for: CD3e-immunotoxin spares CD62Llo Tregs and reshapes organ-specific T-cell composition by preferentially depleting CD3ehi T cells
Source: Front Immunol. 2022 Oct 26;13:1011190. doi: 10.3389/fimmu.2022.1011190 (PMC9643874; doi:10.3389/fimmu.2022.1011190)
Supplement: Supplementary file 1 [file DataSheet_1.docx]

## Supplementary Figures


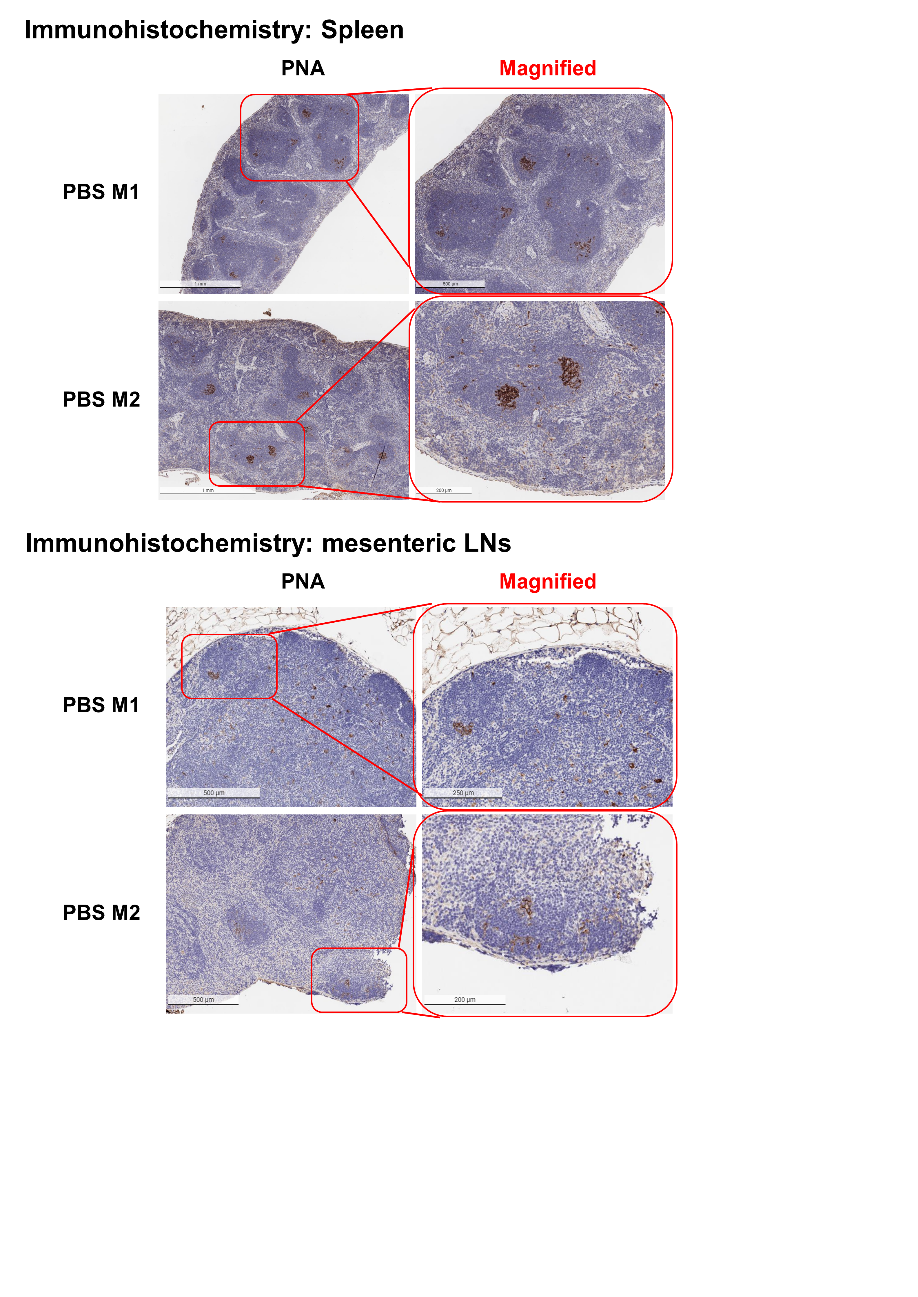

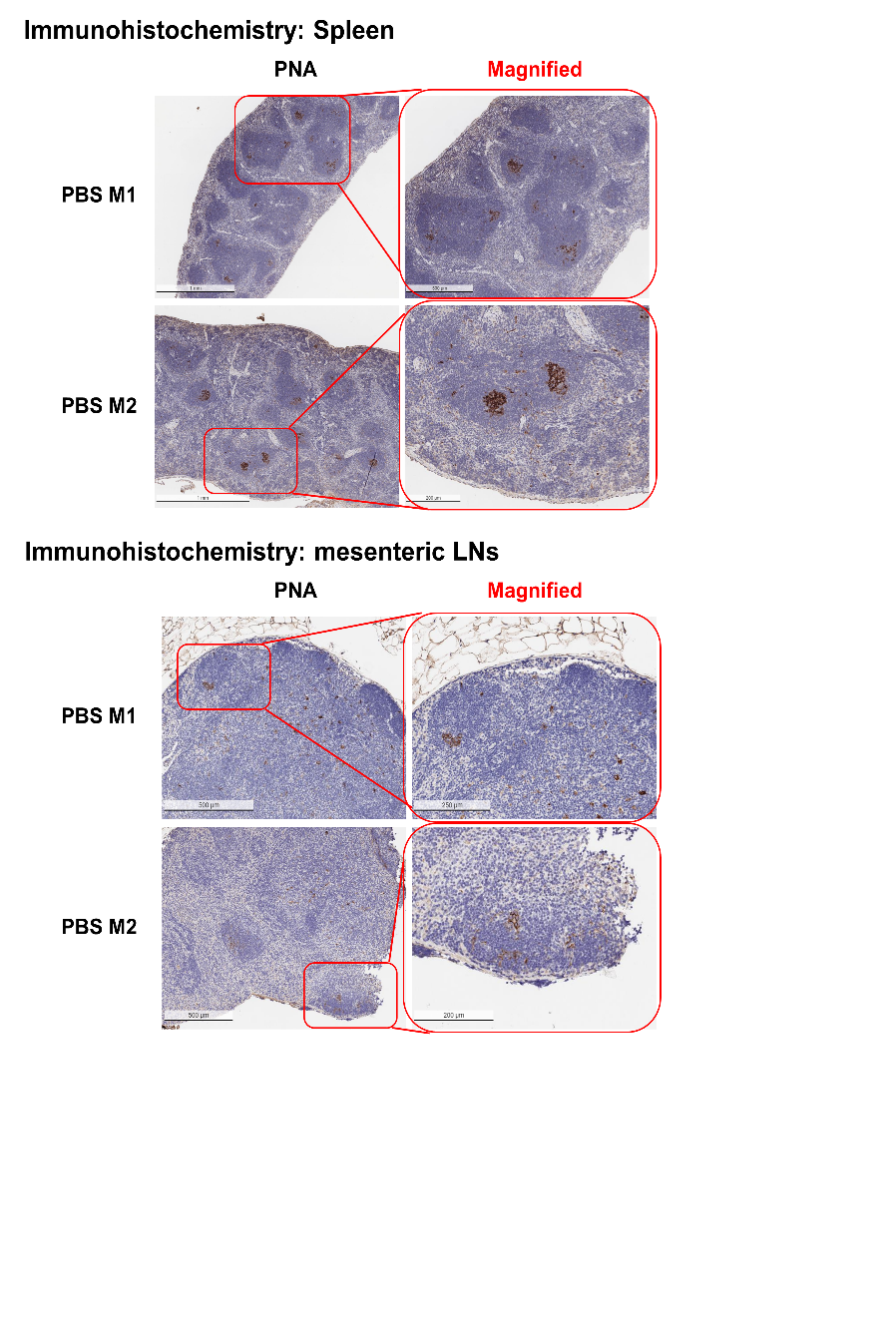


**Supplementary Figure 1.** Formation of the germinal centers following OVA immunization. PNA-positive germinal center follicles are shown for the spleen and mesenteric lymph node (LN) sections from two phosphate-buffered saline (PBS)-treated control mice (PBS M1 and PBS M2) two weeks after the intraperitoneal injection of OVA in complete freund's adjuvant (CFA). Poor GC formation in non-immunized laboratory mice that live in specific pathogen-free (SPF) facilities has been demonstrated previously. (62-64)


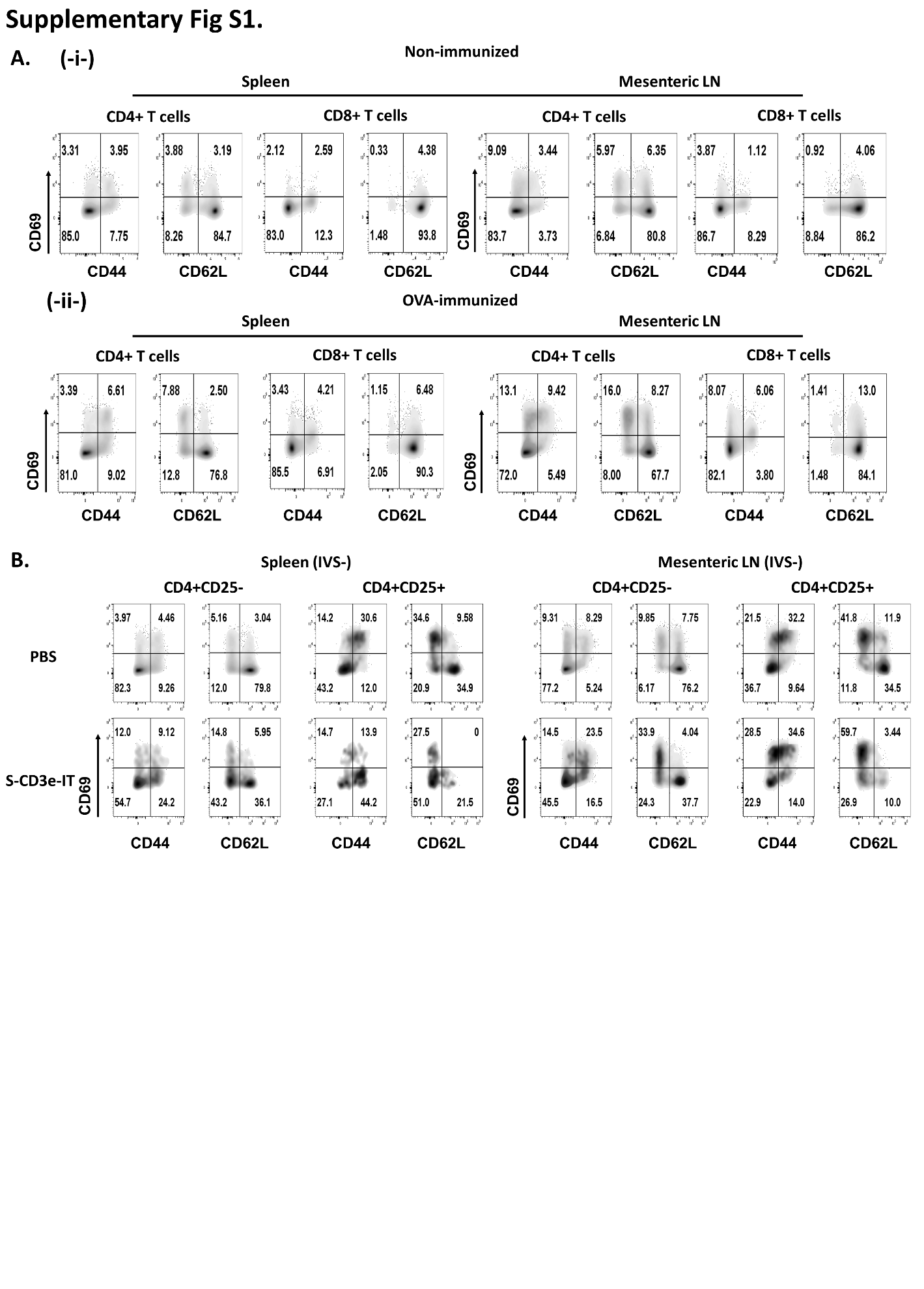


**Supplementary Figure 2.** T-cell activation and infiltration into uninflamed tissues following OVA immunization. (A) Flow image shows the change of CD69+ cells before (i) and after OVA immunization (ii) in spleen and mesenteric LN. The surface CD69 expression (y-axis) is compared on CD44 or CD62L expression (x-axis) for CD4+ T cells (left) and CD8+ T cells (right). CD69 expression was notably increased in LNs cells of OVA-immunized mice compared to non-immunized mice. (B) The percentage change of CD69 expression in CD4+CD25- and CD4+CD25+ cells in tissue-resident (IVS-) spleen (left four panels) and mesenteric LN (right four panels) after S-CD3e-IT treatment. PBS treatment (upper panel) and S-CD3-IT (lower panel) are compared.


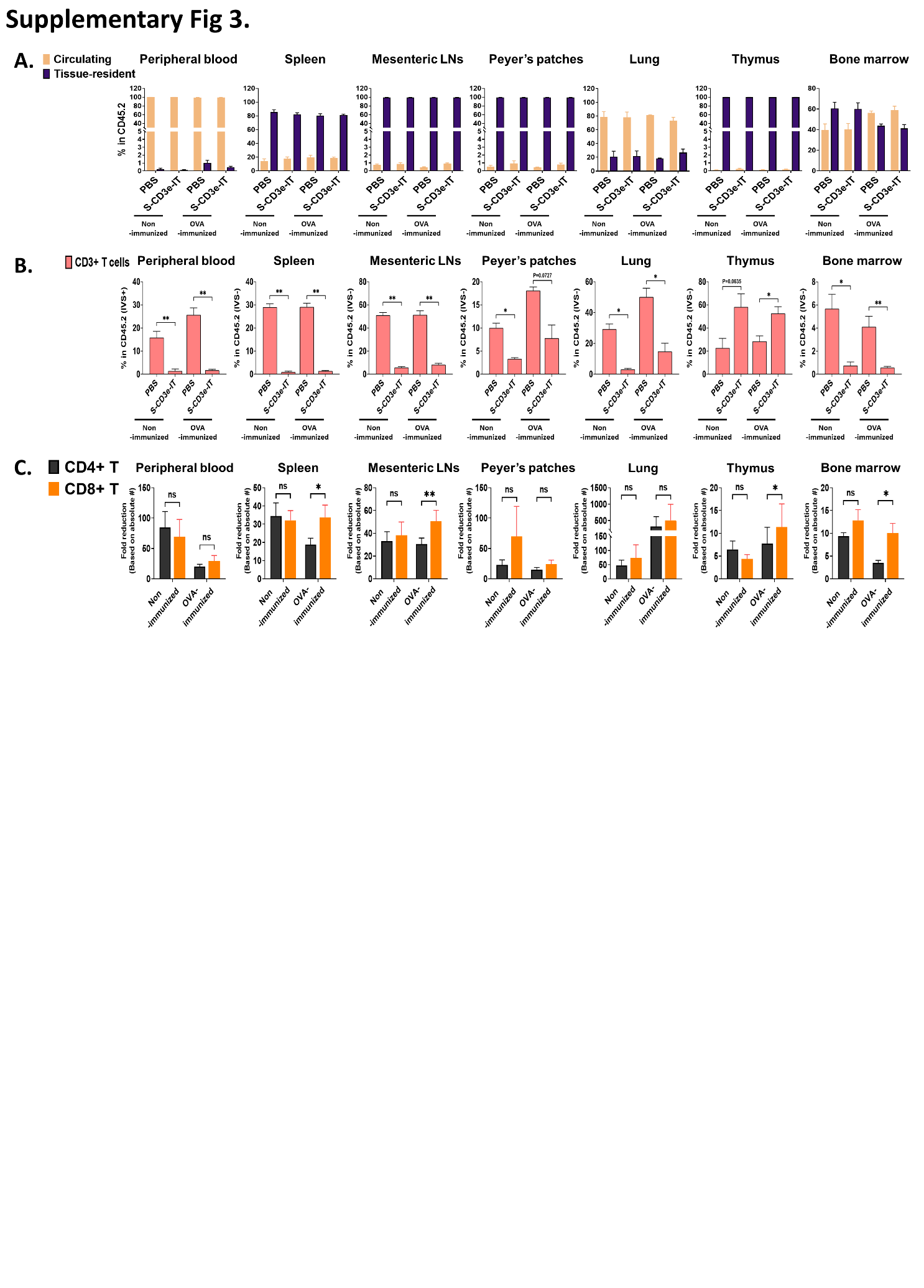


**Supplementary Figure 3.** Circulating and tissue-resident T-cell depletion following S-CD3e-IT treatment. (A) The percentage of circulating (IVS+) and tissue-resident (IVS-) cells, (B) % CD3+ T cells (in CD45.2+ leukocytes, y-axis), and (C) fold-reduction of CD4+ and CD8+ T cells (fold-reduction based on absolute cell counts, y-axis) following S-CD3e-IT treatment are shown for peripheral blood, spleen, mesenteric lymph nodes (LNs), Peyer's patches, lung, Thymus, and bone marrow. Non-immunized mice (PBS; *n* = 4~5 depending on tissues), non-immunized mice (S-CD3e-IT; *n* = 4~6), OVA-immunized mice (PBS; *n* = 3~4), and OVA-immunized mice (S-CD3e-IT; *n* = 7~8), are compared.


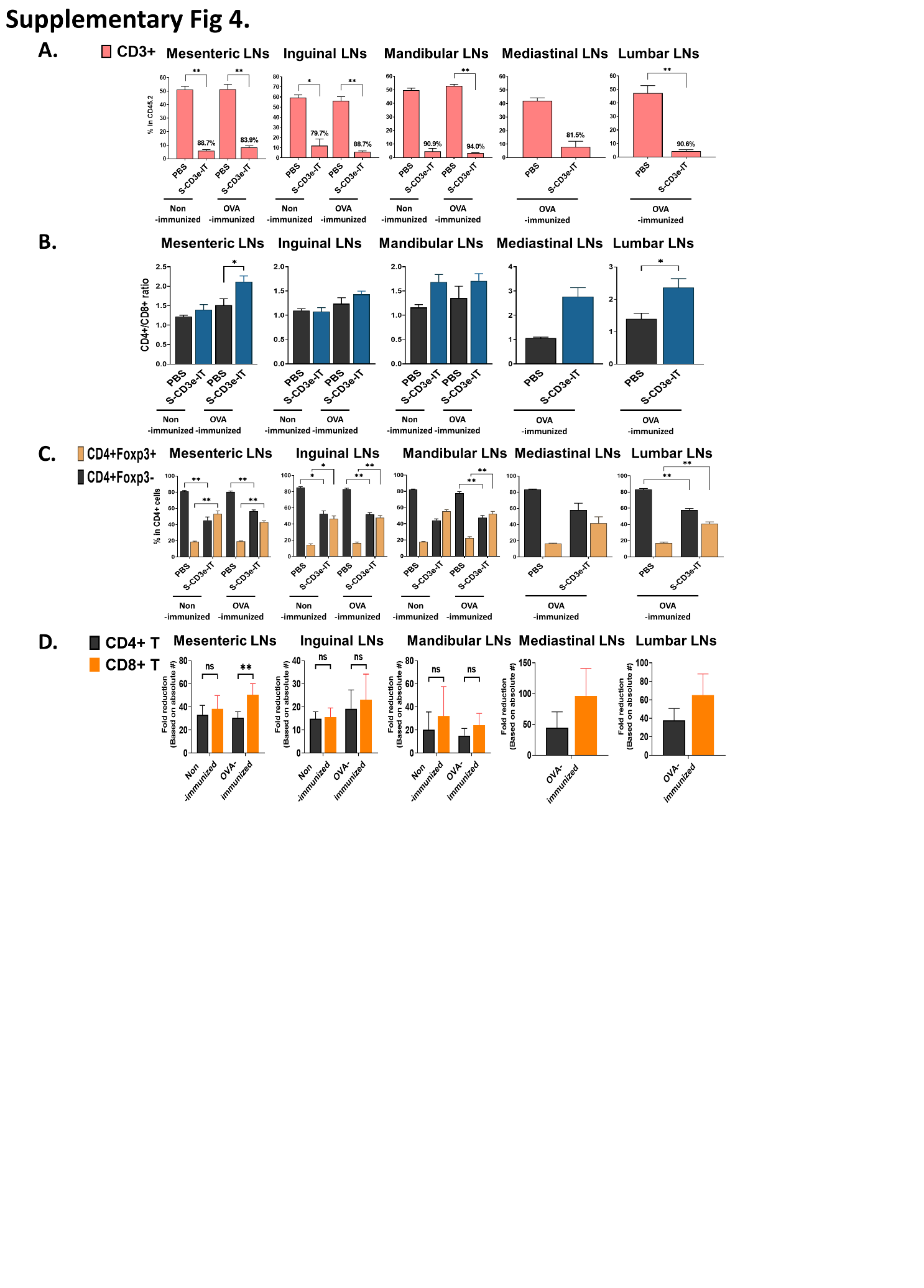


**Supplementary Figure 4**. The effect of S-CD3e-IT on T-cells in different LNs (A) % CD3+ T cells (in CD45.2+ leukocytes, y-axis) are shown for the five LNs (mesenteric, inguinal, mandibular, mediastinal, and lumbar LNs) in different anatomic sites. (B) The ratio of CD4+ to CD8+ is shown for these LNs. This ratio increased following S-CD3e-IT treatment. (C) CD4+Foxp3- and CD4+Foxp3+ (% in CD4+ cells) are shown for these LNs. (D) fold-reduction of CD4+ and CD8+ T cells (fold-reduction based on absolute cell counts, y-axis) following S-CD3e-IT treatment are shown for these LNs. Non-immunized mice (PBS; *n* = 2~4 depending on tissues) non-immunized mice (S-CD3e-IT, *n* = 2~6), OVA-immunized mice (PBS; *n* = 3~4), and OVA-immunized mice (S-CD3e-IT; *n* = 4~8), are compared. (* *p* < 0.05 and ** *p* < 0.01).


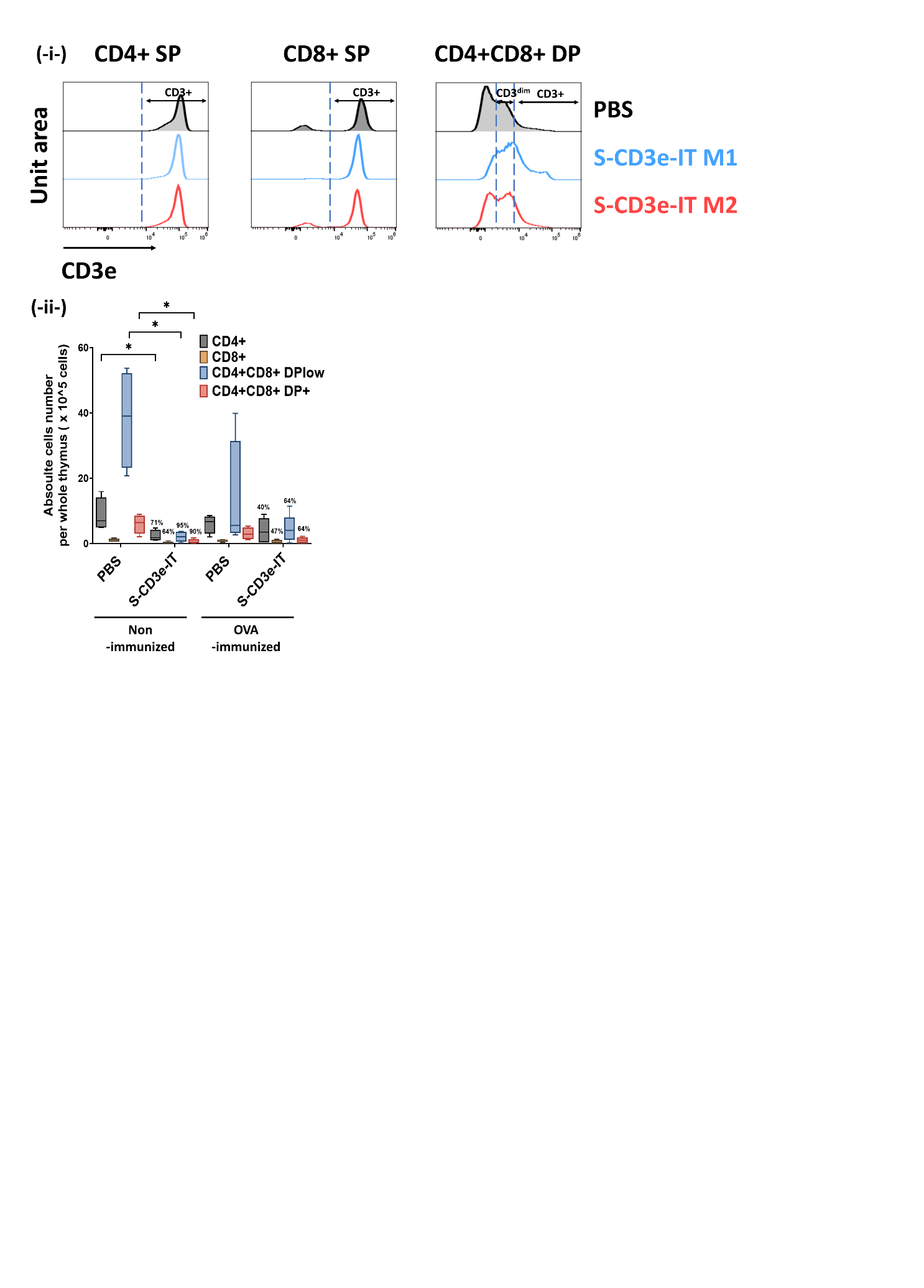


**Supplementary Figure 5.** Differential S-CD3e-IT effects on T subpopulation in the Thymus. **(-i-)** Mean fluorescent intensity (MFI) of CD3e on CD4+, CD8+ single positive (SP), and CD4+CD8+ double positive (DP) cells (**x-axis**) and unit area (**y-axis, upper panel**) are shown for the Thymus. **(-ii-)** Absolute cell count (per the thymus) for CD4+CD3+, CD8+CD3+ SP, CD4+CD8+CD3^lo^, and CD4+CD8+CD3+ cells are shown for the thymus. Cell numbers were calculated based on CountBright Absolute counting beads and the total cell counts per tissue. Non-immunized mice (PBS; *n* = 4 depending on tissues) non-immunized mice (S-CD3e-IT; *n* = 5), OVA-immunized mice (PBS; *n* = 4), and OVA-immunized mice (S-CD3e-IT; *n* = 7), were compared. (* *p* < 0.05).


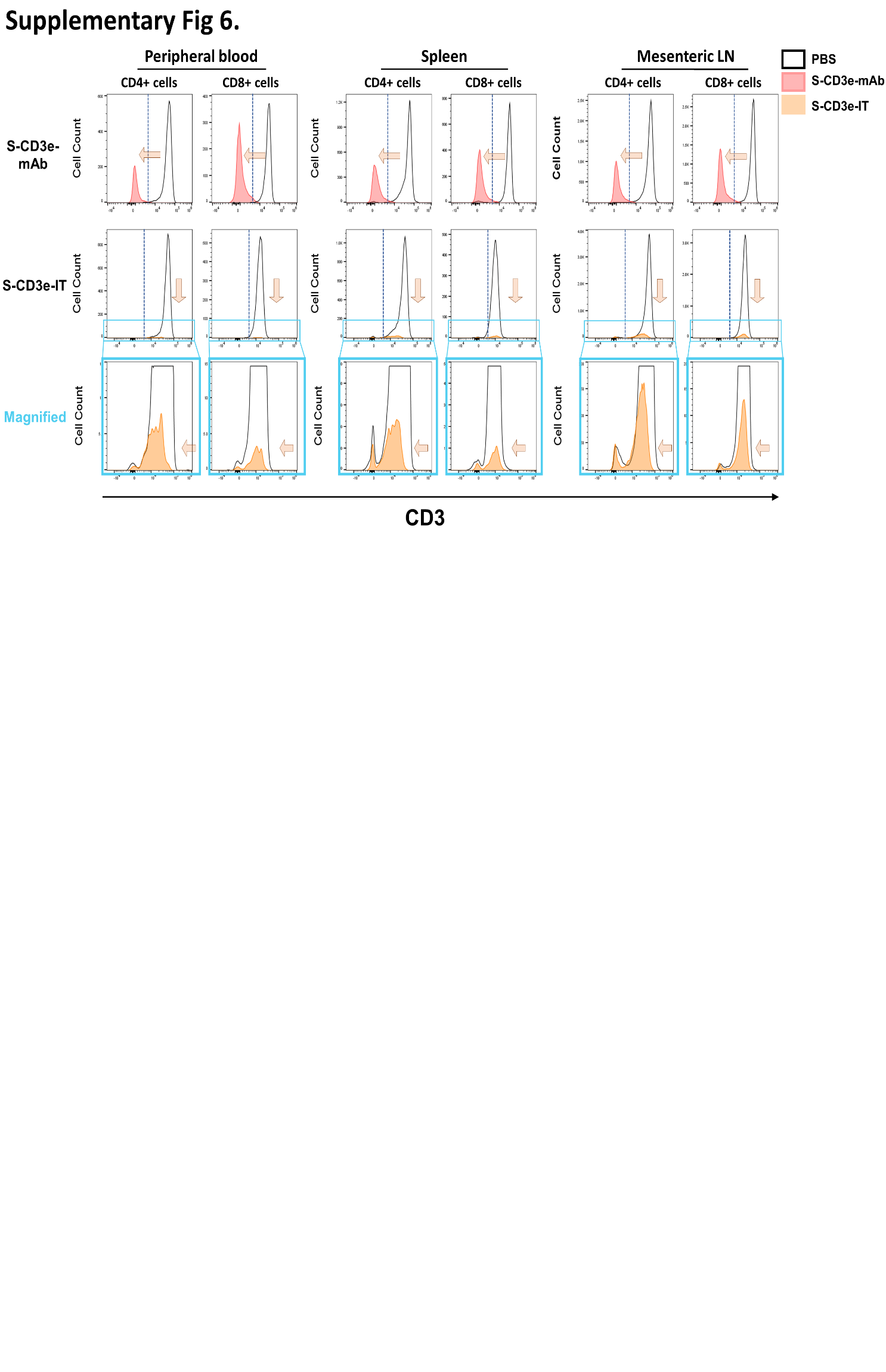


**Supplemental Figure 6.** The distinct mode of action between S-CD3e-mAb and S-CD3e-IT. The mean fluorescent intensity (MFI) of CD3e on the CD4+ and CD8+ cells (x-axis) and cell count (y-axis) is shown for the peripheral blood (left two panels), spleen (middle two panels), and mesenteric LNs (right two panels). Non-mitogenic 145-2C11 (S-CD3e-mAb) almost completely modulated CD3e surface expression on these cells while preserving total T cell numbers; by contrast, S-CD3e-IT showed an effective depletion of T cells with a mild decrease in CD3e MFI on the surviving CD4+ and CD8+ cells (CD3e^dim^ phenotype). Blue boxes represent magnified views of the MFI plot for the S-CD3e-IT group.


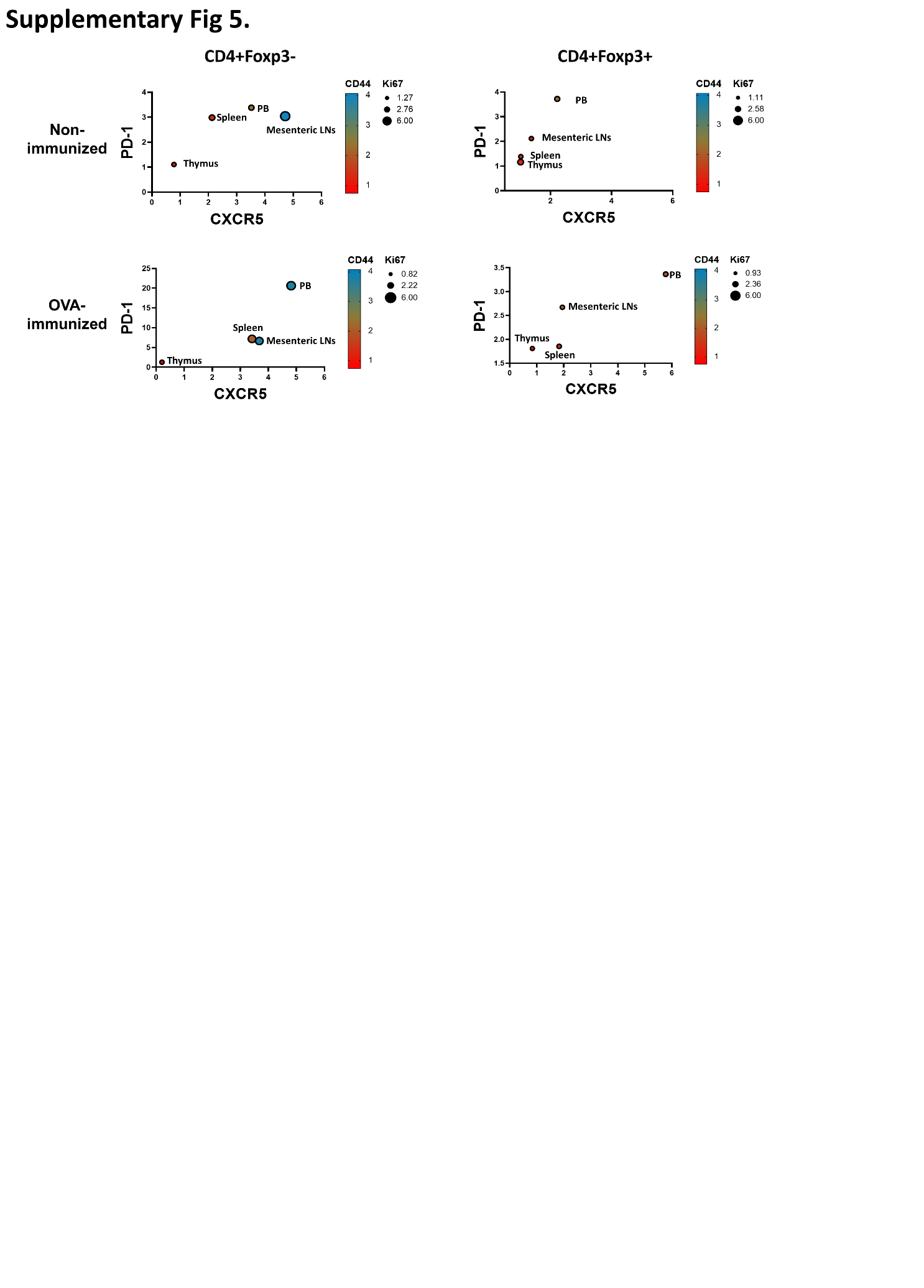


**Supplementary Figure 7.** The fold changes in functional markers of T-cells in different organs. The fold change (S-CD3e-IT / PBS) of CXCR5+ (x-axis), PD-1+ (y-axis), CD44+ (color scheme from red to blue), and Ki67+ markers (circle size) for CD4+Foxp3- (left panels) and CD4+Foxp3+ cells (right panels) are shown for peripheral blood, spleen, mesenteric LN, and thymus. Nonimmunized mice were treated with PBS (*n* = 4~5, depending on organs) or S-CD3e-IT (*n* = 4~6). OVA-immunized mice were treated with PBS (*n* = 3~4) or S-CD3e-IT (*n* = 7~8).
